# Undergoing Diagnostic Evaluation for Possible Cancer Affects the Health-Related Quality of Life in Patients Presenting with Non-Specific Symptoms

**DOI:** 10.1371/journal.pone.0148463

**Published:** 2016-02-03

**Authors:** Ellen Moseholm, Susan Rydahl-Hansen, Bjarne Ørskov Lindhardt

**Affiliations:** 1 Department of Pulmonary and Infectious Diseases, Copenhagen University Hospital, Nordsjælland, Hillerød, Denmark; 2 Research Unit of Clinical Nursing, Bispebjerg and Frederiksberg University Hospital, Copenhagen, Denmark; 3 Department of Public Health, Section for Nursing, Aarhus University, Aarhus, Denmark; 4 Department of Infectious Diseases, Copenhagen University Hospital, Hvidovre, Hvidovre, Denmark; Iranian Institute for Health Sciences Research, ACECR, ISLAMIC REPUBLIC OF IRAN

## Abstract

**Aim:**

Undergoing diagnostic evaluation for possible cancer can affect health-related quality of life (HRQoL). The aims of this study were to examine the HRQoL in patients undergoing a diagnostic evaluation for possible cancer due to non-specific symptoms and further to investigate the impact of socio-demographic and medical factors associated with HRQoL at the time of diagnosis.

**Methods:**

This was a prospective, multicenter survey study that included patients who were referred for a diagnostic evaluation due to non-specific cancer symptoms. Participants completed the EORTC-QLQ-C30 quality of life scale before and after completing the diagnostic evaluation. The baseline and follow-up EORTC-QLQ-C30 scores were compared with reference populations. The impact of socio-demographic and medical factors on HRQoL at follow-up was explored by bootstrapped multivariate linear regression.

**Results:**

A total of 838 patients participated in the study; 680 (81%) also completed follow-up. Twenty-two percent of the patients received a cancer diagnosis at the end of follow-up. Patients presented initially with a high burden of symptoms, less role and emotional functioning and a lower global health/QoL. Most domains improved after diagnosis and no clinically important difference between baseline and follow-up scores was found. Patients reported effects on HRQoL both at baseline and at follow-up compared with the Danish reference population and had similar scores as a cancer reference population. Co-morbidity, being unemployed and receiving a cancer diagnosis had the greatest effect on HRQoL around the time of diagnosis.

**Conclusions:**

Patients with non-specific symptoms reported an affected HRQoL while undergoing a diagnostic evaluation for possible cancer. Morbidity, being unemployed and receiving a cancer diagnosis had the greatest effect on HRQoL around the time of diagnosis.

## Introduction

Early diagnosis is considered a key factor in improving the outcomes of cancer therapy, and long diagnostic intervals have been associated with affected Health-Related Quality of Life (HRQoL) and increased mortality [1–3]. Several countries, including Denmark, have implemented urgent referral Cancer Patient Pathways (CPPs) for patients with a clinical suspicion of cancer [4,5]. The aim is to reduce the length of the diagnostic interval by offering patients optimal diagnosis and treatment opportunities. However, only approximately 40% of all cancer patients seem to have benefited from the implementation of the CPPs [6,7]. This may be because approximately 50% of all cancer patients initially present with vague or non-specific symptoms that may not raise a clinical suspicion of cancer [6,8,9].

A CPP for patients with serious non-specific symptoms and signs of cancer (NSSC-CPP) was therefore introduced in 2012 in Denmark. The objectives are to optimize evaluation and diagnosis, to minimize waiting time and to improve quality of life during the diagnostic work-up phase [5,10]. A clinical coordinator works to optimize logistics, and the aim is to diagnose or refute cancer or any serious illness within 22 days. Patients are, at the time of referral, to be informed about the suspicion of cancer [11].

The diagnostic phase of cancer is differentiated from other cancer stages in that it forms an interface between the suspicion of cancer and the medical confirmations of health/illness status [12]. Although mostly limited to suspicions of a specific cancer illness, previous research suggests that undergoing diagnostic evaluation for possible cancer can affect the HRQoL [13]. In a prospective study, Montezari et al. [14] found a decrease in function and global quality of life during the time of diagnosis in patients with suspected lung cancer. These results were supported by Lheureux et al. [15] and by studies investigating the diagnostic work-up phase of breast cancer [3,16,17] and malignant melanoma [18].

Knowledge about HRQoL in patients undergoing diagnostic evaluation through the urgent referral CPPs implemented in Denmark is limited, specifically in patients suspected of having cancer due to non-specific symptoms not associated with a specific cancer illness. As patients are channeled into the NSSC–CPP program, it provided a unique opportunity to recruit a large population for research about the experience during the diagnostic work-up phase of suspected cancer.

The aims of this study were to examine HRQoL in patients undergoing diagnostic evaluation for possible cancer due to non-specific symptoms and to investigate the impact of socio-demographic and medical factors associated with HRQoL during the diagnostic phase.

## Materials and Methods

### Study population

A prospective, multicenter survey study was conducted between October 1, 2013 and September 30, 2014 at four hospitals in the Capital Region of Denmark. All patients referred to the NSSC-CPP during the study period were eligible to participate in this study. The exclusion criteria were patients younger than 18 years of age, patients with cognitive disorders and patients with language barriers or a referral due to metastasis of an unknown primary tumor.

### Measurements and variables

All participating patients were asked to complete a set of questionnaires prior to diagnosis and again 30 days after referral when the diagnostic evaluation should have been completed. The follow-up questionnaire was sent together with a response envelope via post. Patients not returning the questionnaire received a reminder after two weeks.

The demographic variables were self-reported and included age, gender, marital status, education and employment status. Information on clinical variables (symptoms at referral, duration of symptoms, exposures and smoking) was obtained from the medical records. Information on the diagnoses and co-morbidities, including previous cancer, was collected from the national registries of Statistics Denmark [19]. Co-morbidities were scored according to the Charlson Comorbidity Index [20].

### Health-Related Quality of Life

HRQoL was assessed using the Danish version of the EORTC-QLQ-C30 (version 3.0) quality of life instrument [21]. The EORTC QLQ-C30 is a self-administered questionnaire developed to cover the multi-dimensional concept of HRQoL in cancer patients. The questionnaire consisted of 30 items that aggregated into one global health/QoL scale, five functional scales (physical, emotional, role, cognitive and social functioning), three symptom scales (fatigue, pain and nausea/vomiting) and six single items assessing financial impact and various symptoms. Each item was answered with a four-point scale, from 1 (not at all) to 4 (very much), except for the global health/QoL items, which had seven response options ranging from 1 (very poor) to 7 (excellent) [21,22]. The raw score of each scale/single item was linearly transformed according to the manual to a 0–100 scale [22]. A high score for the global health/QoL scale and functioning scales represented a high/healthy level of QoL and functioning. Conversely, a high score for a symptom scale represented a high level of symptomatology/problems. Missing items were imputed by the methods advocated by the EORTC QLQ research group [22]. Differences in mean scores of 10 or more were regarded as clinically significant [23]. At both baseline and follow-up, participants were asked whether they completed the questionnaire before or after knowledge of their diagnosis.

### Reference sample

Danish population-based reference data were used to compare the EORTC-QLQ-C30 scores with normative scores from the general population [24]. Direct comparisons can be misleading unless age and gender are considered [25]. The reference sample included 1,832 individuals, 47.7% males, with a mean age of 58.3 years (SD 18.7), and thus represented a nearly equal age and gender distribution (Table 1). The EORTC-QLQ-C30 scores were also compared with the EORCT reference cancer population, which consisted of 23,553 international patients with different cancer diagnoses at different stages [26]. The age and gender distributions in this reference sample were likewise similar to the distribution in the study population [26].

**Table 1:** Baseline characteristics.

	Enrolled	Consent only	Not Enrolled	*p-value*
	838	289	1044	
**Age, mean (SD), years**	63.6 (13.5)	60.5 (17.1)	64.7 (16.4)	<0.001*
**Gender, n (%), women**	443 (53)	162 (56)	591 (57)	0.25
**Symptoms at referral, n (%)**				
Weight loss	294 (35)	111 (38)	346 (33)	0.23
Pain	122 (15)	57 (20)	161 (15)	0.11
Suspicion of major illness/cancer	127 (15)	5 (2)	30 (3)	**<0.001**
Abnormal blood tests	106 (13)	36 (12)	118 (11)	0.63
Fatigue	105 (13)	40 (14)	151 (14)	0.48
Pathological lymph node	72 (9)	24 (8)	71 (7)	0.31
Anemia	71 (8)	28 (10)	74 (7)	0.28
Feeling ill	41 (5)	16 (6)	34 (3)	0.09
Night sweats	46 (5)	20 (7)	43 (4)	0.12
Loss of appetite/nausea	35 (4)	25 (9)	45 (4)	0.01*
Fever	34 (4)	10 (3)	34 (3)	0.65
Abdominal disorder	31 (4)	18 (6)	43 (4)	0.18
Increased contact to health system	2 (0.2)	1 (0.3)	0	0.48
Recurrent deep venous thrombosis	1 (0.1)	1 (0.3)	0	0.62
Increased use of medication	0	0	0	
Other	177 (21)	72 (25)	90 (9)	<0.001**
**Cancer diagnosis, n (%)**	188 (22)	35 (12)		**<0.001**
**Co-morbidity**				
0	393 (47)			
1	258 (31)			
≥2	187 (22)			
**Duration of symptoms, weeks, median (IQR)**	12 (6–26)			
Missing, n,%	168 (20)			
**Exposures, n (%)**	158 (19)			
Missing, n,%	5 (0.6)			
**Smoking, n (%)**				
Never smoked	368 (44)			
Former smoker	221 (26)			
Smoker	208 (25)			
Missing, n,%	41 (5)			
**Marital status, n (%)**				
Married/co-inhabitant	565 (67)			
Separated/divorced	97 (12)			
Widow/widower	92 (11)			
Unmarried/single	80 (9)			
Missing, n,%	9 (0.5)			
**Education, n (%)**				
Compulsory <12 years	178 (21)			
Short <15 years/skilled worker	285 (34)			
Medium academic/trade	234 (28)			
Long academic/university level	131 (16)			
Missing, n,%	10 (1)			
**Occupation, n (%)**				
Employed	311 (37)			
Retired/Disability pay	491 (59)			
Unemployed	31 (7)			
Missing, n,%	5 (0.6)			
**Cancer in family, n (%)**	162 (19)			
Missing, n,%	20 (2)			
**Previous cancer in patient**	72 (9)			
Missing, n,%	7 (1)			

*Significant difference between enrolled and consent only

**Significant difference between enrolled and not enrolled

### Ethics

All participants signed an informed consent form before data collection commenced. Approval from the National Committee on Health Research Ethics was not required (H-3-2013-061). The study was approved by The Danish Data Protection Agency (HIH-2013-034). The baseline characteristics for non-participants were obtained anonymously for dropout analyses.

### Statistical Analysis

Categorical variables are described as counts (%), and continuous variables are described as the mean (SD) or medians with the 25^th^ to 75^th^ interquartile range (IRQ), as appropriate. Comparisons between baseline and follow-up EORTC-QLQ-C30 scores and the study population and reference population scores are presented with the difference and the 95% confidence interval of the difference. The impact of socio-demographic and medical factors on role functioning, emotional functioning, fatigue, nausea and vomiting, appetite loss, pain and global QoL scores at follow-up was explored by bootstrapped multivariate linear regression with 2,000 repetitions. Clinically relevant variables associated with HRQoL were included in the model: baseline score, age, gender, cancer diagnosis (yes/no), duration of symptoms (weeks), previous cancer in the patient, co-morbidities (0, 1 or ≥2), marital status, education, occupation and time when the questionnaire was completed at follow-up (before or after diagnosis). As this was a multicenter study, the hospital site was also included as an independent variable in the multivariate analysis. All tests were two-sided, with the significance level set at p<0.05. Analyses were performed using STATA 13.

## Results

### Patient characteristics

**Fig 1:**
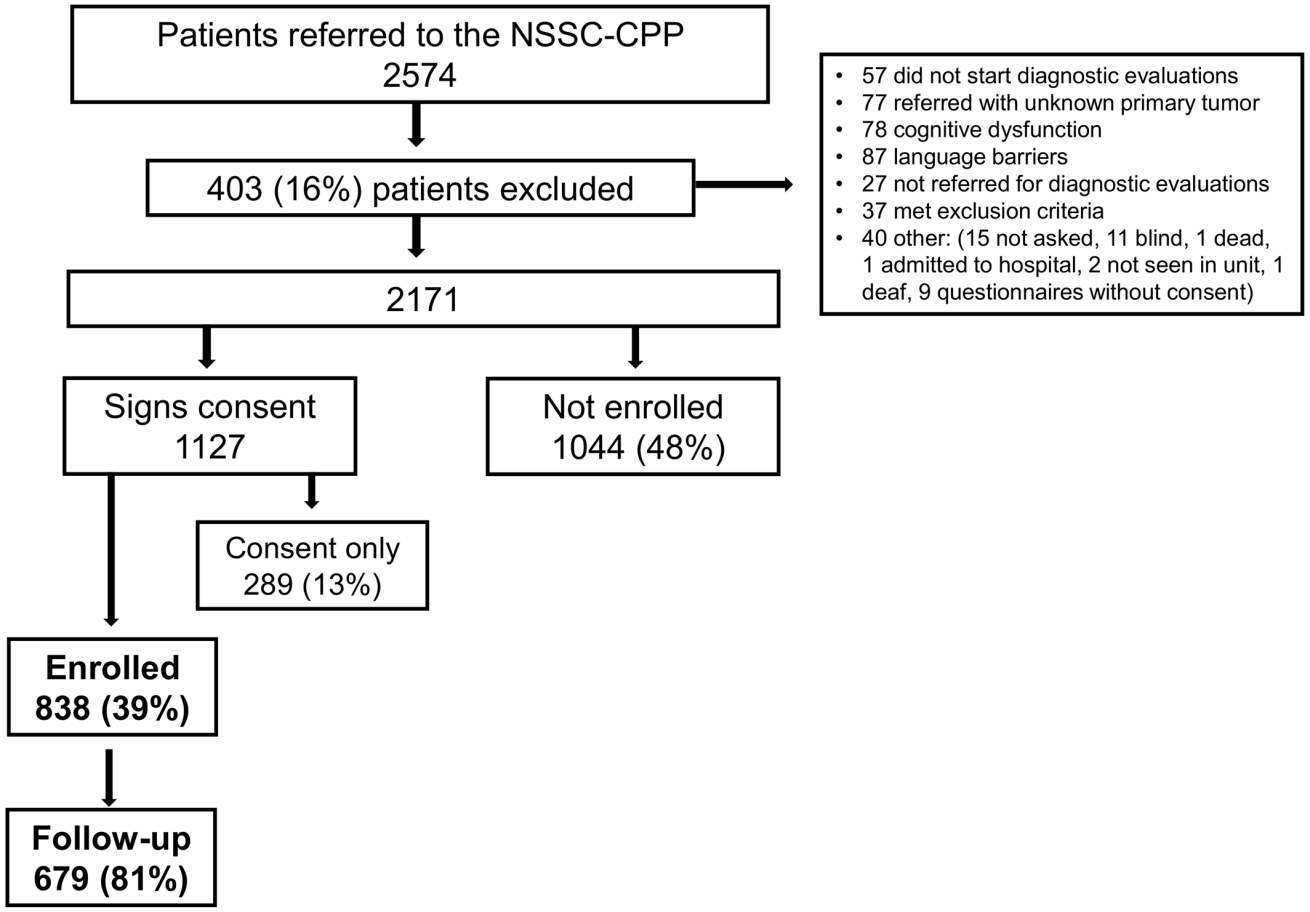
Flowchart.

In total, 2,574 patients were referred to the NSSC-CPP during the study period; 403 patients were initially excluded and 1,044 (48%) patients did not want to participate (‘Not enrolled’). Of the 1,127 patients who signed an informed consent form, 289 (13%) never completed the questionnaire (‘Consent only’), while 838 (39%) returned a completed questionnaire and were thus enrolled in the study (‘Enrolled’). A total of 679 (81%) participating patients completed follow-up (Fig 1). There was no difference in the presence of a cancer diagnosis between the patients who did complete and those who did not complete follow-up. Patients enrolled in the study were significantly older than the ‘consent only’ patients. There was no difference in age between the ‘enrolled’ and ‘not enrolled’ patients. Enrolled patients were less likely to report symptoms of nausea and vomiting at referral than ‘consent only’ patients and were more likely to report other vague symptoms or being referred with “suspicion of cancer” than ‘not enrolled’ patients. There was no difference in gender or symptoms at referral across the groups. Enrolled patients were more likely to be diagnosed with cancer than ‘consent only’ patients. Diagnosis was not available for the ‘not enrolled’ patients (Table 1).

The baseline characteristics of enrolled patients are also presented in Table 1. The mean age was 63.6 years, and 53% of the enrolled patients were women. The most common symptoms at referral were weight loss, pain, abnormal blood work, suspicion of cancer and other non-specific symptoms and fatigue. Twenty-two percent of the enrolled patients received a cancer diagnosis. The most common cancer diagnoses were lymphoma (26, 14%), pulmonary cancer (24, 13%), colorectal cancer (19, 10%) and prostate cancer (16, 9%). More than half of the participants had one or more co-morbidities.

### HRQoL compared with the reference groups

**Fig 2:**
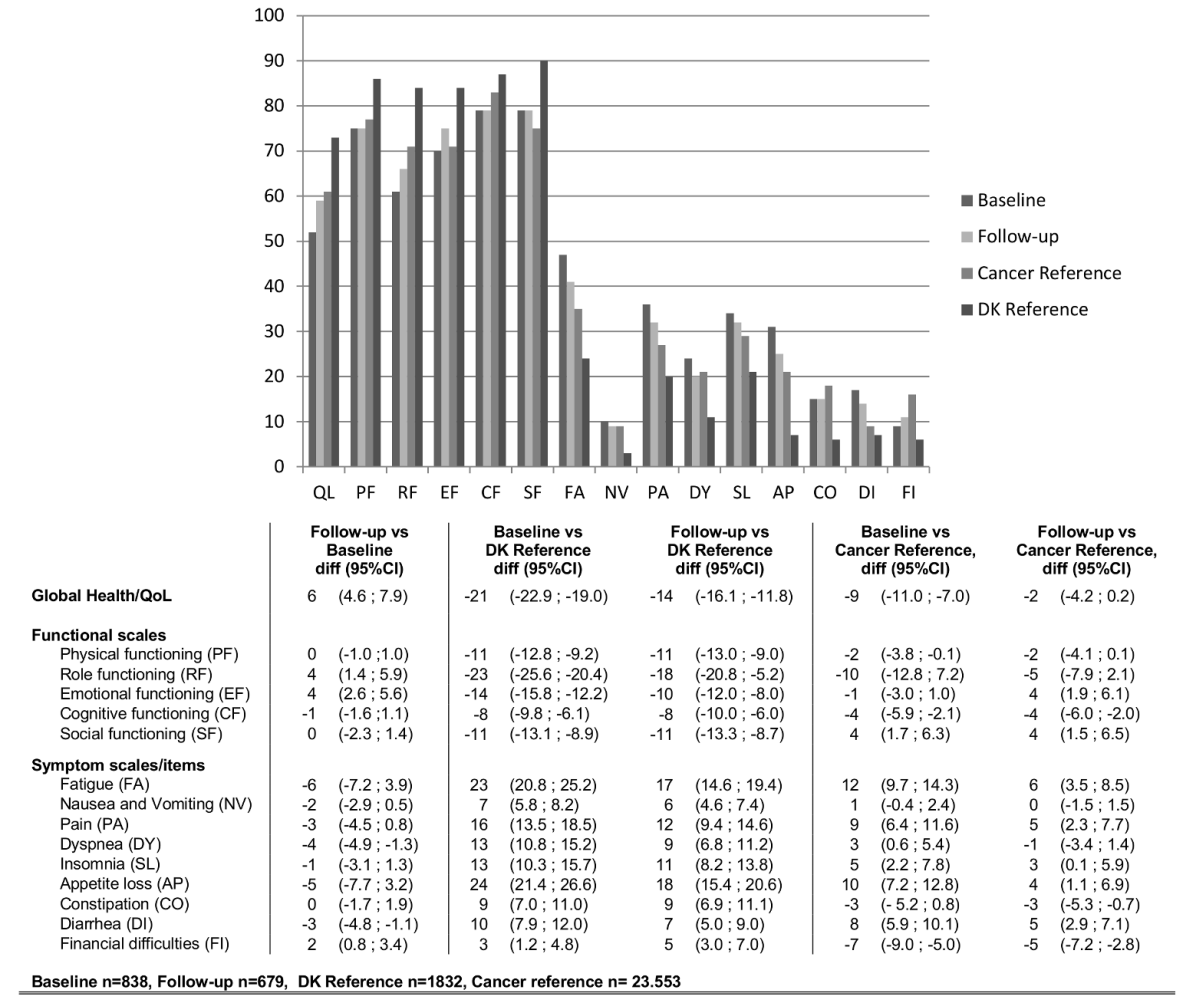
Difference in EORTC-QLQ C30 scores at baseline and follow-up and compared with a Danish reference group and a cancer reference group.

The differences in the EORTC-QLQ-C30 scores at baseline and follow-up and between the study population and reference groups are presented in Fig 2. Participating patients improved in their role and emotional functioning and global QoL scores at follow-up compared with baseline, while there was a slight non-significant deterioration in cognitive functioning. There was no difference in physical functioning and social functioning between baseline and follow-up. Overall, participating patients experienced fewer symptoms at follow-up compared with baseline. On all scales, the difference was less than 10 points.

Compared with a Danish reference group, the participating patients experienced significantly less functioning and global QoL and a higher burden of symptoms at baseline. The difference in scores was less after completing diagnostic evaluations (follow-up). Participating patients scored less on the functional scales and the global QoL scale and higher on the symptoms scales at baseline, compared with the EORTC cancer reference population. At follow-up, this difference was unchanged or less between the groups across the different domains.

### Factors associated with HRQoL during diagnostic evaluation

The results of the multivariate analysis are presented in Tables 2 and 3. Receiving a cancer diagnosis was significantly associated with less role functioning and more fatigue and appetite loss. A longer duration of symptoms was marginally associated with lower emotional functioning. A co-morbidity score of one was significantly associated with increased nausea and vomiting and pain and less global QoL, while a co-morbidity score of ≥2 was associated with less role functioning and global QoL and increased fatigue, pain and nausea and vomiting. Being unmarried was associated with more role functioning and less fatigue, while being retired was associated with more nausea and vomiting. Being unemployed had a negative impact on role functioning, fatigue and global QoL scores. Patients who had completed the follow-up questionnaire after knowledge of diagnosis scored significantly higher on the emotional functioning scale. Overall, the impact of the different variables was small and being unemployed, having a co-morbidity score of ≥2 and receiving a cancer diagnosis had the greatest effect across the different domains. Gender, education and previous cancer did not have an impact on HRQoL in the multivariate analysis. Including the hospital site and time of completion at baseline did not significantly change the estimates in the analysis. The R-square for the different models varied between 0.33 (nausea and vomiting) and 0.52 (fatigue).

**Table 2:** Bootstrapped multivariate regression analysis for role functioning (RF), emotional functioning (EF) and global quality of life (QoL).

	RF			EF			QoL		
	Coeff	95% CI	*p*	Coeff	95% CI	*p*	Coeff	95% CI	*p*
Intercept	27.97	(9.09; 46.85)	0.004	16.06	(2.19; 29.94)	0.02	16.71	(3.39; 30.03)	0.01
**Baseline**	0.58	(0.51; 0.65)	**<0.001**	0.67	(0.59; 0.75)	**<0.001**	0.61	(0.54; 0.68)	**<0.001**
**Age**	-0.01	(-0.26; 0.25)	0.96	0.10	(-0.09; 0.29)	0.31	0.22	(0.03; 0.40)	**0.02**
**Women**	1.10	(-3.47; 5.67)	0.64	0.71	(-2.57; 3.98)	0.67	0.66	(-2.63; 3.97)	0.39
**Cancer**	-8.50	(-16.10; -0.90)	**0.02**	-2.71	(-7.22; 1.78)	0.24	-2.55	(-8.08; 2.97)	0.37
**Duration of symptom (weeks)**	-0.06	(-0.19; 0.08)	0.42	-0.10	(-0.19; -0.01)	**0.03**	-0.09	(-0.19; 0.12)	0.08
**Previous cancer in patient**	2.96	(-6.73; 12.64)	0.55	-0.75	(-7.21; 5.71)	0.82	0.77	(-5.70; 7.24)	0.82
**Co-morbidity**									
0		1			1			1	
1	-5.84	(11.81; 0.13)	0.06	-3.04	(-6.88; 0.81)	0.44	-5.83	(-10.01; -1.65)	**0.006**
≥2	-10.07	(-16.98; -3.17)	**0.004**	1.56	(-8.31; 0.76)	0.48	-6.93	(-11.67; -2.19)	**0.004**
**Marital status**									
Married/Co-inhabitant		1			1			1	
Separated/divorced	4.02	(-3.82; 11.86)	0.32	-2.34	(-8.58; 3.90)	0.46	-1.70	(-7.68; 4.29)	0.58
Widow/widower	3.05	(-6.26; 12.36)	0.52	-0.61	(-6.04; 4.82)	0.83	-1.44	(-8.09; 5.21)	0.67
Unmarried/single	8.50	(0.56; 16.44)	**0.04**	1.48	(-4.31; 7.27)	0.62	1.46	(-4.88; 7.80)	0.65
**Education**									
Basic school/high school		1			1			1	
Vocational training (10–12 years)	2.56	(-3.98; 9.09)	0.44	1.55	(-2.76; 5.86)	0.48	4.43	(-0.61; 9.47)	0.09
Medium academic/trade (<15 years)	1.73	(-4.84; 8.31)	0.61	2.10	(-2.40; 6.61)	0.36	4.30	(-0.69; 9.29)	0.09
Academic/university level (>15 years)	1.28	(-6.71; 9.25)	0.75	4.83	(-0.72; 10.38)	0.08	1.11	(-4.98; 7.21)	0.72
**Occupation**									
Employed		1			1			1	
Unemployed	-14.47	(-27.41; -1.54)	**0.03**	-1.25	(-12.68; 10.10)	0.83	-12.43	(-19.87; -5.01)	**0.001**
Retired/disability/early retirement	2.34	(-4.44; 9.12)	0.50	1.46	(-3.03, 5.95)	0.52	-3.63	(-8.56; 1.30)	0.15
**Follow-up after diagnosis**	2.24	(-3.48; 7.96)	0.44	6.23	(3.49; 9.96)	**0.001**	3.97	(-0.20; 8.13	0.06

**Table 3:** Bootstrapped multivariate regression analysis for fatigue (FA), nausea and vomiting (NV), appetite loss (AP) and pain (PA).

	FA			NV			AP			PA		
	Coeff	95% CI	*p*	Coeff	95% CI	*p*	Coeff	95% CI	*p*	Coeff	95% CI	*p*
Intercept	19.75	(5.80; 33.70)	0.006	7.48	(-1.83; 16.79)	0.12	18.59	(0.69; 36.50)	0.04	26.21	(10.17; 42.24)	0.001
**Baseline**	0.65	(-0.46; -0.05)	**<0.001**	0.47	(0.36; 0.58)	**<0.001**	0.56	(0.49; 0.63)	**<0.001**	0.63	(0.56; 0.60)	**<0.001**
**Age**	-0.25	(-046; -0.05)	**0.02**	-0.17	(-0.32; -0.02)	**0.03**	-0.25	(-0.50; 0.001)	**0.05**	-0.33	(-0.55; -0.10)	**0.004**
**Women**	1.40	(-2.03; 4.83)	0.42	1.61	(-0.82; 4.04)	0.19	1.92	(-2.57; 6.42)	0.40	1.79	(-2.15; 5.74)	0.37
**Cancer**	5.88	(0.43; 11.35)	**0.04**	3.35	(-0.43; 7.13)	0.08	10.06	(2.95; 17.17)	**0.006**	-0.89	(-7.71; 5.93)	0.80
**Duration of symptom (weeks)**	0.04	(-0.06; 0.14)	0.46	0.001	(-0.06; 0.06)	0.99	0.11	(-0.02; 0.25)	0.10	-0.01	(-0.12; 0.11)	0.94
**Previous cancer in patient**	-0.71	(-8.05; 6.62)	0.85	-0.59	(-4.91; 3.73)	0.78	5.52	(-4.21; 15.25)	0.27	-4.83	(-12.79; 3.12)	0.23
**Co-morbidity**												
0		1			1			1			1	
1	4.11	(-0.24; 8.46)	0.06	3.27	(0.37; 6.16)	**0.03**	3.39	(-2.36; 9.14)	0.25	9.23	(3.91; 14.55)	**0.001**
≥2	8.97	(3.66; 14.28)	**0.001**	4.64	(1.38; 7.89)	**0.005**	4.53	(-2.33; 11.39)	0.20	8.34	(2.51; 14.18)	**0.005**
**Marital status**												
Married/Co-inhabitant		1			1			1			1	
Separated/divorced	0.49	(-5.58; 6.57)	0.87	4.18	(-1.25; 9.61)	0.13	2.40	(-5.79; 10.60)	0.57	3.05	(-3.55; 9.65)	0.37
Widow/widower	0.77	(-5.98; 7.52)	0.82	-0.95	(-5.63; 3.74)	0.69	1.76	(-5.52; 9.03)	0.64	-2.19	(-9.59; 5.21)	0.56
Unmarried/single	-6.60	(-12.71; -0.49)	**0.03**	-1.35	(-5.28; 2.57)	0.50	-3.78	(-12.85; 5.29)	0.42	-4.81	(-12.27; 2.65)	0.21
**Education**												
Basic school/high school		1			1			1			1	
Vocational training (10–12 years)	-0.36	(-5.08; 4.36)	0.88	-1.68	(-4.87; 1.49)	0.30	-0.38	(-6.84; 6.09)	0.91	1.72	(-4.06; 7.49)	0.56
Medium academic/trade (<15 years)	-2.84	(-7.77; 2.09)	0.26	-0.84	(-3.94; 2.27)	0.60	-3.64	(-9.88; 2.60)	0.25	1.46	(-4.51; 7.42)	0.63
Academic/university level (>15 years)	-3.15	(-9.01; 2.69)	0.29	0.18	(-3.74; 4.10)	0.92	-4.22	(-11.85; 3.41)	0.28	-1.44	(-8.62; 5.75)	0.70
**Occupation**												
Employed		1			1			1			1	
Unemployed	9.91	(0.84; 18.98)	**0.03**	2.90	(-6.31; 12.11)	0.54	1.82	(-13.73; 17.37)	0.82	6.20	(-4.55; 16.94)	0.26
Retired/disability/early retirement	1.56	(-3.60; 6.73)	0.55	3.78	(0.05; 7.51)	**0.05**	4.94	(-1.31; 11.19)	0.12	1.65	(-4.59; 7.88)	0.61
**Follow-up after diagnosis**	-2.02	(-6.14; 2.11)	0.34	-1.38	(-4.52; 4.04)	0.19	-3.90	(-9.81; 2.02)	0.20	-4.54	(-9.74; 0.67)	0.09

## Discussion

### Main findings

This study investigated HRQoL in patients undergoing diagnostic evaluation for possible cancer due to non-specific symptoms not associated with a specific cancer illness. Our results showed that the patients experienced an affected HRQoL while undergoing diagnostic evaluations for possible cancer. Role functioning, emotional functioning and global QoL was especially affected, but improved after completing diagnostic evaluations.

Studies investigating HRQoL around the time of a cancer diagnosis have found that functioning and symptoms deteriorated after diagnosis [14,15,18]. These studies only included patients with a known cancer diagnosis. Knowledge of a cancer diagnosis has been shown to have a negative impact especially on physical and role functioning [27]. In our study sample, 78% did not have cancer, and patients improved on almost all symptom scales. This is likely to have had an effect on their functioning and global QoL [26].

Although population reference data for the EORTC-QLQ-C30 have been published for several European countries [24,28–33], differences between countries have been reported [24,30,34]. Hence, in this study, we used a recent study reporting population-based data from a large random sample from the Danish population with similar age and gender distributions [24]. Compared with this reference population, patients referred to the NSSC-CPP had worse functioning and a higher burden of symptoms both at baseline and follow-up. Patients also reported worse functioning and a higher burden of symptoms, especially fatigue and appetite loss, at baseline compared with a cancer reference population [26]. Although these differences diminished over time, the follow-up scores were similar to those of a cancer population, indicating that HRQoL is affected even after diagnosis. The cancer reference population consisted of more than 23,000 cancer patients with different cancer diagnoses and at different stages. The main source of data was from cancer clinical trials and epidemiological studies and was based on pre-treatment QoL data only [26]. Patients included in this reference dataset were probably early in their disease trajectory with uncertainty in relation to treatment and prognosis. Role functioning seems to be particularly affected in patients referred to the NSSC-CPP compared with the cancer reference population, and this could be associated with the high burden of symptoms and the uncertainty related to unknown diagnoses.

The incidence of the most common cancers found in our study was similar to those in the cancer reference population, except for lymphoma, which included only 2% of the cancer reference population vs. 14% in the study population [26]. Patients diagnosed with lymphoma may present with fever symptoms than other cancer patients [35], and this could have an impact on their experience of HRQoL. However, we found no difference in global QoL at either baseline or follow-up between patients diagnosed with lymphoma and patients diagnosed with other types of cancer illness (data are not shown).

A recent study found that patients referred to the NSSC-CPP consisted of a very heterogeneous group presenting with over 80 different symptoms[36]. Similar to other studies, we found weight loss, pain and abnormal blood tests to be the most common symptoms at referral [36]. Although more than 10% of the patients were referred to the NSSP-CPP due to abnormal blood tests, most of these patients were not asymptomatic, as 78% were also experiencing other symptoms such as weight loss, fatigue and pain (data are not shown).

Co-morbidity, being unemployed and receiving a cancer diagnosis had the greatest effect on HRQoL around the time of diagnosis. Having two or more co-morbidities was significantly associated with less functioning and less global QoL and a higher burden of symptoms, and this is similar to population-based findings [24,37]. It is also well known that HRQoL may be affected by several other socio-demographic factors, such as unemployment, as seen in our study [24]. Receiving a cancer diagnosis was associated with less functioning and higher symptom scores. Similar results have been shown in patients diagnosed with lung cancer and breast cancer [3,15]. The patients diagnosed with cancer were most likely to be early in their cancer treatment at the time of follow-up and therefore were still affected, not only by symptoms but also by the novelty and insecurity of the situation. Patients who completed the follow-up questionnaire prior to knowledge of diagnosis scored significantly higher on the emotional functioning scale. This highlights the impact of insecurity on HRQoL, and further research is needed to explore how patients can best be supported during this time. Interestingly, the baseline score and the duration of symptoms were the only other variables that were significantly associated with emotional functioning at follow-up. However, the impact was almost non-existent when looking at the scores. A clinical coordinator is associated with the NSSC-CPP to optimize logistics and to ensure that the patients were well informed throughout the diagnostic evaluations. Whether this had an impact on patients’ emotional well-being needs to be determined.

Overall, the impact of the included variables was small. Furthermore, the multivariate models explained only between 33% and 52% of the variance. Other factors, such as anxiety and coping, may therefore have an important impact on HRQoL, and this should be examined in future studies. Further research is also needed to explore any long-term psychological implications of going through diagnostic evaluations for possible cancer.

### Strengths and limitations of the study

A major strength of this study was the prospective, multicenter design with a cohort of consecutive patients. Patients were encouraged to complete the baseline questionnaire prior to knowledge of their diagnosis. However, as patients were experiencing symptoms and were informed about the suspicion of cancer at baseline, this may not render a true baseline measurement of HRQoL. We compared the EORTC-QLQ-C30 results with both a general reference population and a cancer reference population to provide support during the interpretation of the results. Diagnosis and co-morbidity data were collected via the National Patient Registry, which is considered to be precise and valid [19].

A main limitation might be the selection bias caused by a low response rate; only 39% of eligible patients participated. A low response rate does not necessarily indicate non-response bias [38,39], as the differences between enrolled patients and non-participating patients were small. However, we do not know whether non-participating patients were experiencing more or less co-morbidity, social problems or anxiety than participating patients, and selection bias could therefore have been introduced.

We found no clinical difference in the EORTC-QLQ-C30 scores between baseline and follow-up, which could indicate that follow-up was collected too close to the diagnostic phase. Only four patients had a prior history of anxiety; thus, a prior history of anxiety was not included in the multivariate analysis due to the small numbers.

Patients´ experiences when undergoing diagnostic evaluation for cancer due to non-specific symptoms have not, to our knowledge, been described previously. The results from this study showed that HRQoL was affected during diagnostic evaluations. Patients presented initially with a high burden of symptoms, less role and emotional functioning, and a lower global health/QoL. Most domains improved after diagnosis. Morbidity, being unemployed and receiving a cancer diagnosis had the greatest effect on HRQoL around the time of diagnosis.

## Supporting Information

S1 Dataset (XLS)

## References

[1] Torring ML, Frydenberg M, Hansen RP, Olesen F, Vedsted P. Evidence of increasing mortality with longer diagnostic intervals for five common cancers: a cohort study in primary care. Eur J Cancer. 2013;49: 2187–2198. 10.1016/j.ejca.2013.01.025 23453935

[2] Brocken P, van der Heijden EH, Oud KT, Bootsma G, Groen HJ, Donders AR, et al Distress in suspected lung cancer patients following rapid and standard diagnostic programs: a prospective observational study. Psychooncology. 2015;24: 433–441. 10.1002/pon.3660 25201175

[3] Harcourt D, Ambler N, Rumsey N, Cawthom SJ. Evaluation of a one-stop breast lump clinic: a randomized controlled trial. 1998;7: 314–319.

[4] Olesen F, Hansen RP, Vedsted P. Delay in diagnosis: the experience in Denmark. Br J Cancer. 2009;101 Suppl 2: S5–8. 10.1038/sj.bjc.6605383 19956163 PMC2790711

[5] Probst HB, Hussain ZB, Andersen O. Cancer patient pathways in Denmark as a joint effort between bureaucrats, health professionals and politicians—a national Danish project. Health Policy. 2012;105: 65–70. 10.1016/j.healthpol.2011.11.001 22136810

[6] Jensen H, Torring ML, Olesen F, Overgaard J, Vedsted P. Cancer suspicion in general practice, urgent referral and time to diagnosis: a population-based GP survey and registry study. BMC Cancer. 2014;14: 636 10.1186/1471-2407-14-636 25175155 PMC4164756

[7] Meechan D, Gildea C, Hollingworth L, Richards MA, Riley D, Rubin G. Variation in use of the 2-week referral pathway for suspected cancer: a cross-sectional analysis. Br J Gen Pract. 2012;62: e590–7. 10.3399/bjgp12X654551 22947579 PMC3426597

[8] Nielsen TN, Hansen RP, Vedsted P. Symptom presentation in cancer patients in general practice. Ugeskr Laeger. 2010;172: 2827–2831. 20961502

[9] Neal RD, Din NU, Hamilton W, Ukoumunne OC, Carter B, Stapley S, et al Comparison of cancer diagnostic intervals before and after implementation of NICE guidelines: analysis of data from the UK General Practice Research Database. Br J Cancer. 2014;110: 584–592. 10.1038/bjc.2013.791 24366304 PMC3915139

[10] Jensen H, Torring ML, Olesen F, Overgaard J, Fenger-Gron M, Vedsted P. Diagnostic intervals before and after implementation of cancer patient pathways—a GP survey and registry based comparison of three cohorts of cancer patients. BMC Cancer. 2015;15: 308 10.1186/s12885-015-1317-7 25900050 PMC4412104

[11] Sundhedsstyrelsen. Diagnostisk pakkeforløb for patienter med uspecifikke symptomer på alvorlig sygdom, der kunne være kræft. Version: 3.0; Versionsdato: 27. juni 2012 ed. Kbh.; 2012.

[12] Poole K. The emergence of the 'waiting game': a critical examination of the psychosocial issues in diagnosing breast cancer. J Adv Nurs. 1997;25: 273–281. 9044000 10.1046/j.1365-2648.1997.1997025273.x

[13] Brocken P, Prins JB, Dekhuijzen PN, van der Heijden HF. The faster the better?-A systematic review on distress in the diagnostic phase of suspected cancer, and the influence of rapid diagnostic pathways. Psychooncology. 2012;21: 1–10. 10.1002/pon.1929 22905349

[14] Montazeri A, Milroy R, Hole D, McEwen J, Gillis CR. How quality of life data contribute to our understanding of cancer patients' experiences? A study of patients with lung cancer. Qual Life Res. 2003;12: 157–166. 12639062 10.1023/a:1022232624891

[15] Lheureux M, Raherison C, Vernejoux JM, Nguyen L, Nocent C, Tunon De Lara M, et al Quality of life in lung cancer: does disclosure of the diagnosis have an impact? Lung Cancer. 2004;43: 175–182. 14739038 10.1016/j.lungcan.2003.08.018

[16] Montazeri A, Vahdaninia M, Harirchi I, Ebrahimi M, Khaleghi F, Jarvandi S. Quality of life in patients with breast cancer before and after diagnosis: an eighteen months follow-up study. BMC Cancer. 2008;8: 330-2407-8-330. 10.1186/1471-2407-8-330 PMC2588619 19014435

[17] Stover AM, Mayer DK, Muss H, Wheeler SB, Lyons JC, Reeve BB. Quality of life changes during the pre- to postdiagnosis period and treatment-related recovery time in older women with breast cancer. Cancer. 2014;120: 1881–1889. 10.1002/cncr.28649 24647996 PMC4047201

[18] Al-Shakhli H, Harcourt D, Kenealy J. Psychological distress surrounding diagnosis of malignant and nonmalignant skin lesions at a pigmented lesion clinic. J Plast Reconstr Aesthet Surg. 2006;59: 479–486. 16749193 10.1016/j.bjps.2005.01.010

[19] Lynge E, Sandegaard JL, Rebolj M. The Danish National Patient Register. Scand J Public Health. 2011;39: 30–33. 10.1177/1403494811401482 21775347

[20] Charlson ME, Pompei P, Ales KL, MacKenzie CR. A new method of classifying prognostic comorbidity in longitudinal studies: development and validation. J Chronic Dis. 1987;40: 373–383. 3558716 10.1016/0021-9681(87)90171-8

[21] Aaronson NK, Ahmedzai S, Bergman B, Bullinger M, Cull A, Duez NJ, et al The European Organization for Research and Treatment of Cancer QLQ-C30: a quality-of-life instrument for use in international clinical trials in oncology. J Natl Cancer Inst. 1993;85: 365–376. 8433390 10.1093/jnci/85.5.365

[22] Fayers P, Aaronson N, Bjordal K, Groenvold M, Curran D, Bottomley A. The EORTC QLQ-C30 Scoring Manual (3rd Edition). 2001;3rd edition.

[23] Osoba D, Rodrigues G, Myles J, Zee B, Pater J. Interpreting the significance of changes in health-related quality-of-life scores. J Clin Oncol. 1998;16: 139–144. 9440735 10.1200/JCO.1998.16.1.139

[24] Juul T, Petersen MA, Holzner B, Laurberg S, Christensen P, Gronvold M. Danish population-based reference data for the EORTC QLQ-C30: associations with gender, age and morbidity. Qual Life Res. 2014;23: 2183–2193. 10.1007/s11136-014-0675-y 24676897

[25] Hjermstad MJ, Fayers PM, Bjordal K, Kaasa S. Using reference data on quality of life—the importance of adjusting for age and gender, exemplified by the EORTC QLQ-C30 (+3). Eur J Cancer. 1998;34: 1381–1389. 9849421 10.1016/s0959-8049(98)00136-1

[26] Scott, NW. et al. on behalf of the EORTC Quality of Life Group. EORTC QLQ-C30 Reference Values Manual. 2008.

[27] Montazeri A, Hole DJ, Milroy R, McEwen J, Gillis CR. Does knowledge of cancer diagnosis affect quality of life? A methodological challenge. BMC Cancer. 2004;4: 21 15151702 10.1186/1471-2407-4-21 PMC420242

[28] van de Poll-Franse LV, Mols F, Gundy CM, Creutzberg CL, Nout RA, Verdonck-de Leeuw IM, et al Normative data for the EORTC QLQ-C30 and EORTC-sexuality items in the general Dutch population. Eur J Cancer. 2011;47: 667–675. 10.1016/j.ejca.2010.11.004 21134739

[29] Michelson H, Bolund C, Nilsson B, Brandberg Y. Health-related quality of life measured by the EORTC QLQ-C30—reference values from a large sample of Swedish population. Acta Oncol. 2000;39: 477–484. 11041109 10.1080/028418600750013384

[30] Schwarz R, Hinz A. Reference data for the quality of life questionnaire EORTC QLQ-C30 in the general German population. Eur J Cancer. 2001;37: 1345–1351. 11435063 10.1016/s0959-8049(00)00447-0

[31] Hjermstad MJ, Fayers PM, Bjordal K, Kaasa S. Health-related quality of life in the general Norwegian population assessed by the European Organization for Research and Treatment of Cancer Core Quality-of-Life Questionnaire: the QLQ = C30 (+ 3). J Clin Oncol. 1998;16: 1188–1196. 9508207 10.1200/JCO.1998.16.3.1188

[32] Derogar M, van der Schaaf M, Lagergren P. Reference values for the EORTC QLQ-C30 quality of life questionnaire in a random sample of the Swedish population. Acta Oncol. 2012;51: 10–16. 10.3109/0284186X.2011.614636 21961499

[33] Klee M, Groenvold M, Machin D. Quality of life of Danish women: population-based norms of the EORTC QLQ-C30. Qual Life Res. 1997;6: 27–34. 9062439 10.1023/a:1026461310761

[34] Fayers PM. Interpreting quality of life data: population-based reference data for the EORTC QLQ-C30. Eur J Cancer. 2001;37: 1331–1334. 11435060 10.1016/s0959-8049(01)00127-7

[35] Mounter PJ, Lennard AL. Management of non-Hodgkin's lymphomas. Postgrad Med J. 1999;75: 2–6. 10396578 10.1136/pgmj.75.879.2 PMC1741115

[36] Ingeman ML, Christensen MB, Bro F, Knudsen ST, Vedsted P. The Danish cancer pathway for patients with serious non-specific symptoms and signs of cancer-a cross-sectional study of patient characteristics and cancer probability. BMC Cancer. 2015;15: 421 10.1186/s12885-015-1424-5 25990247 PMC4445271

[37] Fossa SD, Hess SL, Dahl AA, Hjermstad MJ, Veenstra M. Stability of health-related quality of life in the Norwegian general population and impact of chronic morbidity in individuals with and without a cancer diagnosis. Acta Oncol. 2007;46: 452–461. 17497312 10.1080/02841860601182641

[38] Mealing NM, Banks E, Jorm LR, Steel DG, Clements MS, Rogers KD. Investigation of relative risk estimates from studies of the same population with contrasting response rates and designs. BMC Med Res Methodol. 2010;10: 26 10.1186/1471-2288-10-26 20356408 PMC2868856

[39] Choung R, Locke G, Schleck C, Ziegenfuss J, Beebe T, Zinsmeister A, et al A low response rate does not necessarily indicate non- response bias in gastroenterology survey research: a population-based study. J Public Health. 2013;21: 87–95.

